# Multi-scale data reveal a CD24(+) MUCL1(+) tumor subgroup associated with unfavorable prognosis in ER+ breast cancer

**DOI:** 10.3389/fimmu.2025.1695689

**Published:** 2025-10-10

**Authors:** Yingxi Li, Junming Cao, Linyue Hai, Jing Zeng, Dongchen Tian, Yue Yu, Zhaohui Chen, Yao Tian

**Affiliations:** ^1^ Health Science Center, Ningbo University, Ningbo, Zhejiang, China; ^2^ The First Department of Breast Cancer, Key Laboratory of Cancer Prevention and Therapy, Tianjin’s Clinical Research Center for Cancer, National Clinical Research Center for Cancer, Key Laboratory of Breast Cancer Prevention and Therapy, Tianjin Medical University Cancer Institute and Hospital, Tianjin Medical University, Tianjin, China; ^3^ Department of Thoracic Surgery, The Affiliated LiHuiLi Hospital of Ningbo University, Ningbo, Zhejiang, China

**Keywords:** ER+ breast cancer, MUCL1(+) CD24(+) cells, tumor microenvironment, radiomics, single-cell RNA sequencing

## Abstract

**Objective:**

Breast cancer remains the leading cause of cancer-associated death for women globally. For the group of ER+ breast cancer patients, there are still some problems of poor prognosis that need to be solved. This study aims to identify the poor prognostic tumor subgroups for the prognostic stratification of ER+ patients.

**Methods:**

Through a comprehensive multi-omics strategy, we systematically characterized the biological and clinical significance of MUCL1+CD24+ cells in breast cancer, and we used multiplex immunohistochemistry to confirm the poor role of MUCL1+CD24+ cells.

**Results:**

Single-cell transcriptomics unraveled the cellular ontogeny and immune microenvironment interactions of this subset, while bulk RNA sequencing exposed significant pathway heterogeneity and differential immunotherapy responses associated with varying cellular abundance levels. Genomic landscape analysis pinpointed specific somatic mutations correlated with MUCL1(+) CD24(+) cell infiltration patterns, findings that were subsequently validated through multiplex immunohistochemistry to demonstrate strong prognostic value. Crucially, we developed a clinically translatable radiomics approach that successfully correlated specific MRI features with cellular prevalence, establishing a foundation for noninvasive detection of this aggressive cellular subpopulation.

**Conclusions:**

This integrative approach, spanning molecular to imaging analyses, provides novel insights into both the biological drivers and clinical implications of MUCL1(+) CD24(+) cells in breast cancer progression.

## Introduction

Breast cancer remains the predominant cause of cancer-associated mortality in the female population globally (1). The remarkable molecular heterogeneity of this malignancy results in significantly divergent clinical prognoses among affected individuals (2). Estrogen receptor-positive (ER+) breast cancer represents nearly 70% of all breast malignancies (3). Although the continuous research and development of endocrine therapy and CDK4/6 inhibitors have enabled ER+ breast cancer patients to have a better prognosis, the development of endocrine resistance remains a critical clinical challenge, especially in advanced disease stages (4, 5). Current data indicate that approximately 40%–50% of metastatic ER+ cases acquire treatment resistance within 24 months, frequently resulting in disease progression and diminished survival outcomes (6). Therefore, it is of vital importance to explore the factors of poor prognosis in the group of ER+ breast cancer patients, which is of great significance for the formulation of treatment strategies and the screening of people with poor prognosis.

The emergence of single-cell transcriptomic profiling has revolutionized our understanding of tumor heterogeneity, facilitating the discovery of rare cellular subpopulations that contribute to treatment refractoriness (7). This technological breakthrough enables the correlation of genomic alterations with cell-specific transcriptional programs, offering novel opportunities to decipher resistance mechanisms and discover actionable vulnerabilities (8). Besides that, advanced imaging modalities such as MRI, CT, and PET now play a pivotal role in predicting therapeutic response, molecular subtyping, and prognostic stratification in oncology (9). The integration of radiomic features with multi-omics data offers a transformative approach for discovering clinically relevant biomarkers and personalized treatment strategies.

In this study, we identified a tumor subgroup characterized by high CD24 and MUCL1 expression, which was linked to poor prognosis and invasive behavior. We also uncovered somatic mutations associated with the infiltration of CD24(+) MUCL1(+) cells, along with potential inhibitors for personalized treatment in ER^+^ breast cancer. Furthermore, a radiomic model effectively estimated the infiltration levels of these cells. Overall, our findings offer a new therapeutic target and a non-invasive strategy for immunotherapy and individualized treatment in ER+ breast cancer patients.

## Materials and methods

### Data collection and quality control

A total of 14 single-cell RNA sequencing data including seven ER+ breast cancer and seven paired lymph node metastatic tissues were downloaded from GSE161529 (10). The Seurat (v4.3.3) (11) package is used for single-cell sequencing quality control processes including standardization, clustering, and dimensionality reduction. Rigorous quality control was implemented, including (1) the removal of low-quality cells based on mitochondrial gene content (<20%), unique molecular counts (>500 transcripts/cell), and detected genes (>200 genes/cell). Doubletdfinder (v2.0.4) (12) and harmony packages were utilized to remove the mixed cells and batch effect. Bulk RNA expression profiles were also obtained from TCGA database. Patients with complete clinical information and expression profiles were included in the subsequent analysis. Finally, 474 breast cancer samples with ER status (+), PR status (+/-), and HER2 status (-) were included in the study. To reduce the effect of gene length and depth of sequencing, the format of the matrix was transformed into TPM.

### Downstream analyses of scRNA-seq

A total of 40 cell clusters were identified after strict quality control procedures. The cell types were further annotated based on public research (13). Eight main cell types were annotated, and epithelial cells were isolated for malignant cell identification. The CNV correlation and score were calculated by infercnv (v1.14.2) (14) package. The details could be found in a referenced study (15). Subsequently, the same procedure as previously described was performed on the cluster resolution of malignant cells. The epitools (v0.5-10.1) package was used to investigate the tissue preference of malignant cell subgroups. The top marker genes of each tumor subgroup were analyzed by the “findallmarker” function. The trajectory inference and cell developmental direction of malignant cells were analyzed by using SCP (v0.4.7.9000) and vector packages. Additionally, the dynamic lineages of tumor cells were reconstructed, and gene clusters were further annotated based on biological process.

### Estimation of cell abundance

Since single-cell data cannot directly reflect cell abundance, bulk RNA data was used to infer cell abundance. The single-cell RNA matrix was used as referenced matrix, while the TCGA-BRCA matrix was used as observed matrix. CIBERSORTX (https://cibersortx.stanford.edu/) was utilized to infer cell abundance. The high and low abundance of the C4 subgroup was divided based on the median value of the absolute score of C4. The statistics of survival analysis was investigated by log-rank test.

### Gene set enrichment analysis

The differential genes between high and low abundance of the C4 subgroup was evaluated by using the limma (v3.54.2) (16) package. All significant genes were reordered and analyzed based on hallmark gene sets. *P*-value <0.05 was considered statistically significant.

### Genomic mutation analysis

The TCGA-BRCA somatic mutation data was downloaded by using the tcgabiolinks (v2.26.0) (17) package. Maftools (v2.14.0) (18) package was used to integrate genomic profiles. The differential mutations between the high- and low-C4 groups are identified by the mafCompare function. Fisher’s exact test was used to detect statistical significance. Co-occurrence and co-exclusion patterns between genes were identified by the somatic interactions function.

### TIDE and CMAP analysis

Tumor immune dysfunction and exclusion (TIDE) analysis was conducted by using an online database (http://tide.dfci.harvard.edu/). Concretely, the expression matrix was scaled and uploaded to the TIDE database. The TIDE score of each sample was obtained, and chi-square test was used to detect the statistical differences in immune responses between the high- and low-C4 subgroups. The potential drugs for high-C4 patients were identified by using the CMAP database (19). The details could be found in our previous study.

### Non-invasive radiomics construction

A total of 11 ER+ breast cancers with both molecular subtype information and radiomic imaging profiles were included in the study. Pyradiomics (v3.0.1) was used to extract the radiomics features. Two experienced radiologists with over 10 years of experience in breast oncology together performed fully the manual segmentation of the tumors. All features underwent Z-score normalization, and Pearson correlation was used to filter candidate features correlated with C4 cluster infiltration (*p* < 0.05). LASSO regression algorithm was performed to establish a linear regression model to estimate the C4 cluster abundance. Pearson correlation and ROC curve were used to calculate the association between the abundance of C4 cluster and radiomic score and assess the discriminative efficiency of the model.

### Breast cancer specimens

A total of 30 ER+ breast cancer specimens from patients who underwent surgery were collected at Tianjin Medical University Cancer Institute and Hospital. Informed written consent was obtained from the participants. The study was approved by the Ethical Committee of Tianjin Medical University Cancer Institute and Hospital and adhered to the ethical guidelines of the Helsinki Declaration.

### Multiplexed fluorescent IHC staining and H&E staining

The consecutive ER+ breast cancer tissues were used to evaluate the expression level of EpCAM, MUCL1, and CD24. In brief, 5-μm slides were deparaffinized and rehydrated through a graded series of ethanol solutions prior to antigen retrieval in heated citric acid buffer (pH 6.0). Each slide was put through three sequential rounds of staining, each including a protein block with blocking buffer followed by primary antibody and corresponding secondary HRP-conjugated antibody. Each HRP-conjugated antibody mediated the covalent binding of a different fluorophore for signal amplification. This reaction was followed by additional antigen retrieval in heated citric acid buffer (pH 6.0) in microwave for 15 min to remove the bound antibodies before the next step. After three sequential reactions, the slides were counterstained with DAPI for 10 min and mounted with fluorescence mounting medium. Anti-EPCAM (GB12274, Servicebio), anti-MUCL1 (BS-17247R, Bioss), and anti-CD24 (BS-23867R, Bioss) were used. Images were acquired with a Nikon Eclipse C1 microscope.

For histological examination, a paraffin-embedded ear tissue was cut into 5-μm sections with H&E staining (G1120, Solarbio) and other staining protocols accordingly (G3632 and G3670, Solarbio).

### Statistical analysis

All bioinformatics analyses in this study were based on R studio (v4.2.2) and python 3.7. All statistical methods can be found in the corresponding methods sections.

## Results

### Annotation of cell types in ER+ breast cancer single-cell data

**Figure 1:**
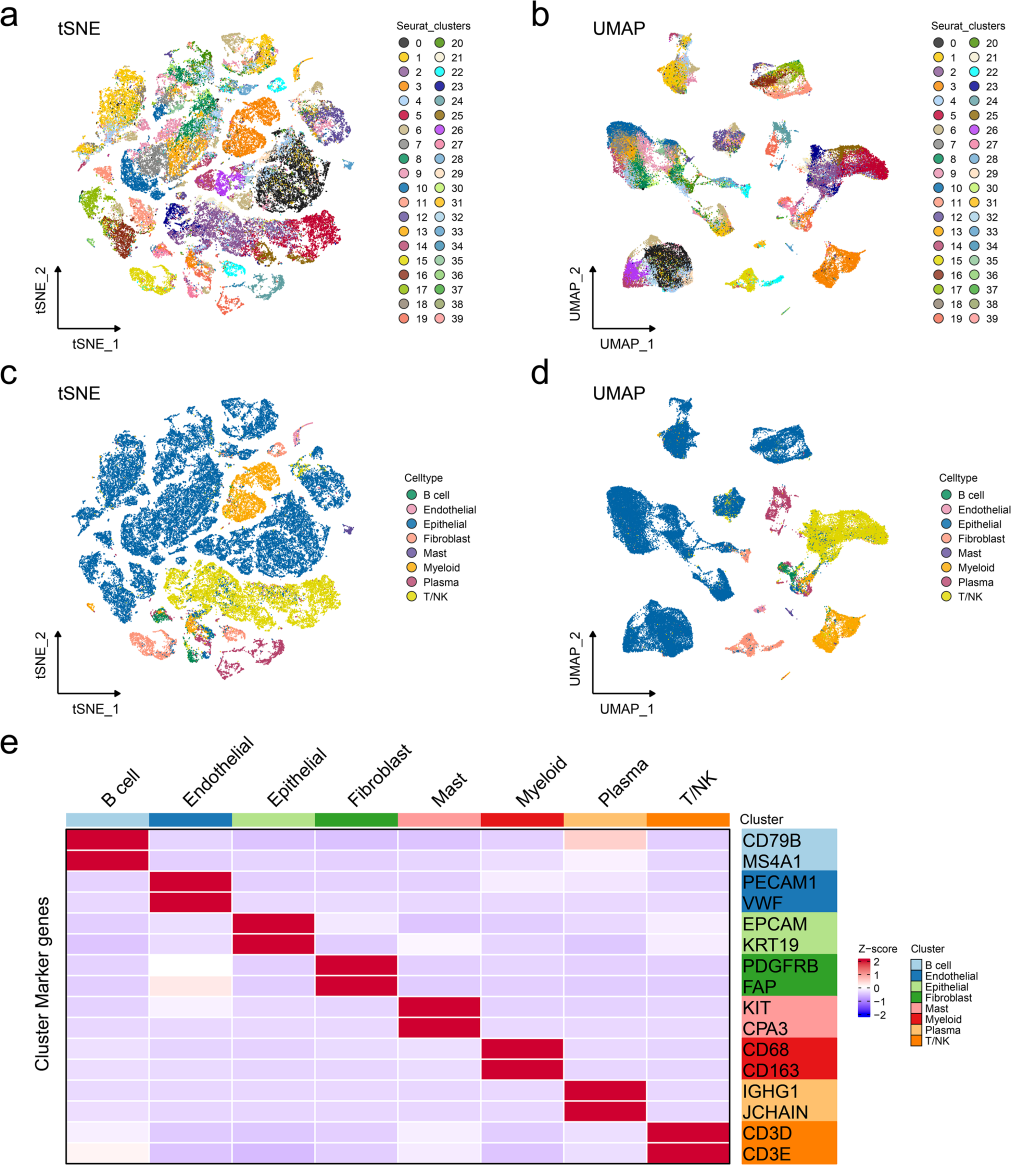
Annotation of cell types in ER+ breast cancer single-cell data. Reduced-dimension visualization of t-distributed stochastic neighbor embedding **(a)** and uniform manifold approximation and projection **(b)** of cell clusters in single-cell RNA datasets, with each color representing a different cell type. Reduced-dimension visualization of t-distributed stochastic neighbor embedding **(c)** and uniform manifold approximation and projection **(d)** of cell types in single-cell RNA datasets, with each color representing a different cell type. **(e)** Heatmap showing the specific markers for cell annotation.

**Figure 2:**
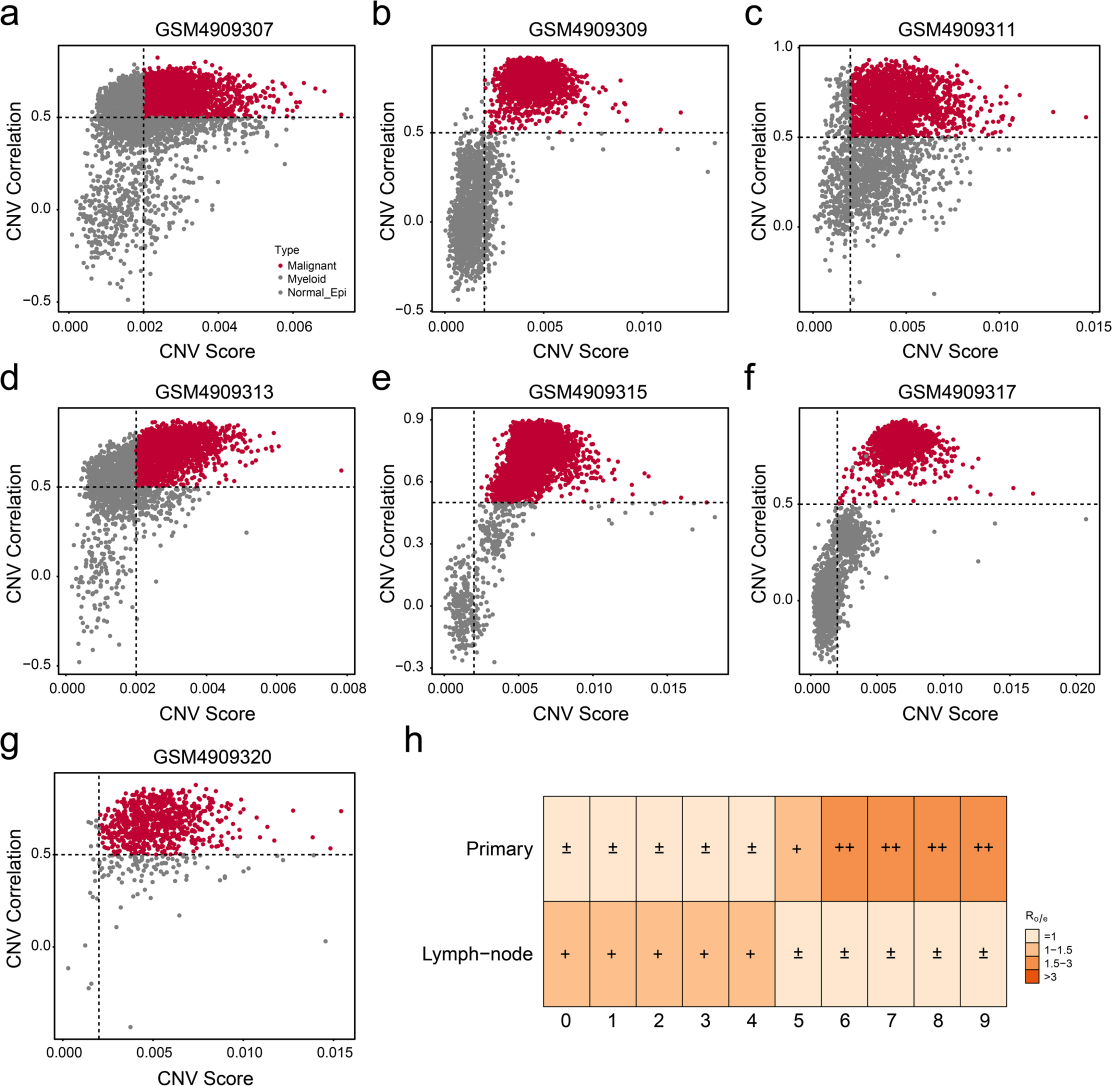
Isolation of malignant cells from epithelial cells. **(a–g)** The copy number variation (CNV) correlation and score of each primary ER+ breast cancer were visualized by scatter plot. **(h)** Tissue preference confirmed the enrichment tendency of each tumor subgroup.

To investigate different cell types in ER+ breast cancer, single-cell transcriptomic analysis was performed on 14 ER+ breast cancer samples from GSE161529, including seven primary ER+ breast cancer tissues and seven paired lymph node metastatic tissues. Rigorous quality control and doublet removal were implemented as previously described. Following rigorous quality control and normalization, a total of 63,369 high-confidence cells were obtained, and 40 distinct cell clusters were identified (**Figures 1A, B**). Dimensionality reduction using uniform manifold approximation and projection and t-distributed stochastic neighbor embedding demonstrated the clear segregation of the annotated cell populations (**Figures 1C, D**). Then, cell type annotation was performed by integrating marker gene expression with reference datasets from online databases. We identified eight major cell populations: B cells (*N* = 817), endothelial cells (*N* = 434), epithelial cells (*N* = 41,860), fibroblasts (*N* = 2444), mast cells (*N* = 340), myeloid cells (*N* = 4433), plasma cells (*N* = 2,520), and T/NK cells (*N* = 10,521) (**Figure 1E**). Standard marker genes were employed for population annotation, including EPCAM (epithelial), PECAM1 (endothelial), and other well-characterized identifiers. In summary, eight main cell types in ER+ breast cancers were identified for the subsequent research. Subsequently, we performed DNA copy number variation analysis to recognize highly confident malignant cells. As shown in **Figures 2A–G**, malignant cells presented obvious high CNV correlation coefficient and CNV score compared to normal epithelial cells and myeloid cells, indicating that the set threshold successfully distinguishes tumor cells from normal cells. The tumor cells were standardized and clustered, and 10 tumor subgroups were identified. Tissue preference analysis showed clusters 5–9 (C5–C9) to be more likely enriched in primary ER+ breast cancer tissues, while clusters 0–4 (C0–C4) were more inclined to be enriched in lymph node metastatic tissues (**Figure 2H**). Taken together, tumor cells were successfully isolated, and 10 distinct tumor subgroups were firstly identified for subsequent analyses.

### The cell trajectory and heterogeneity in tumor microenvironment

**Figure 3:**
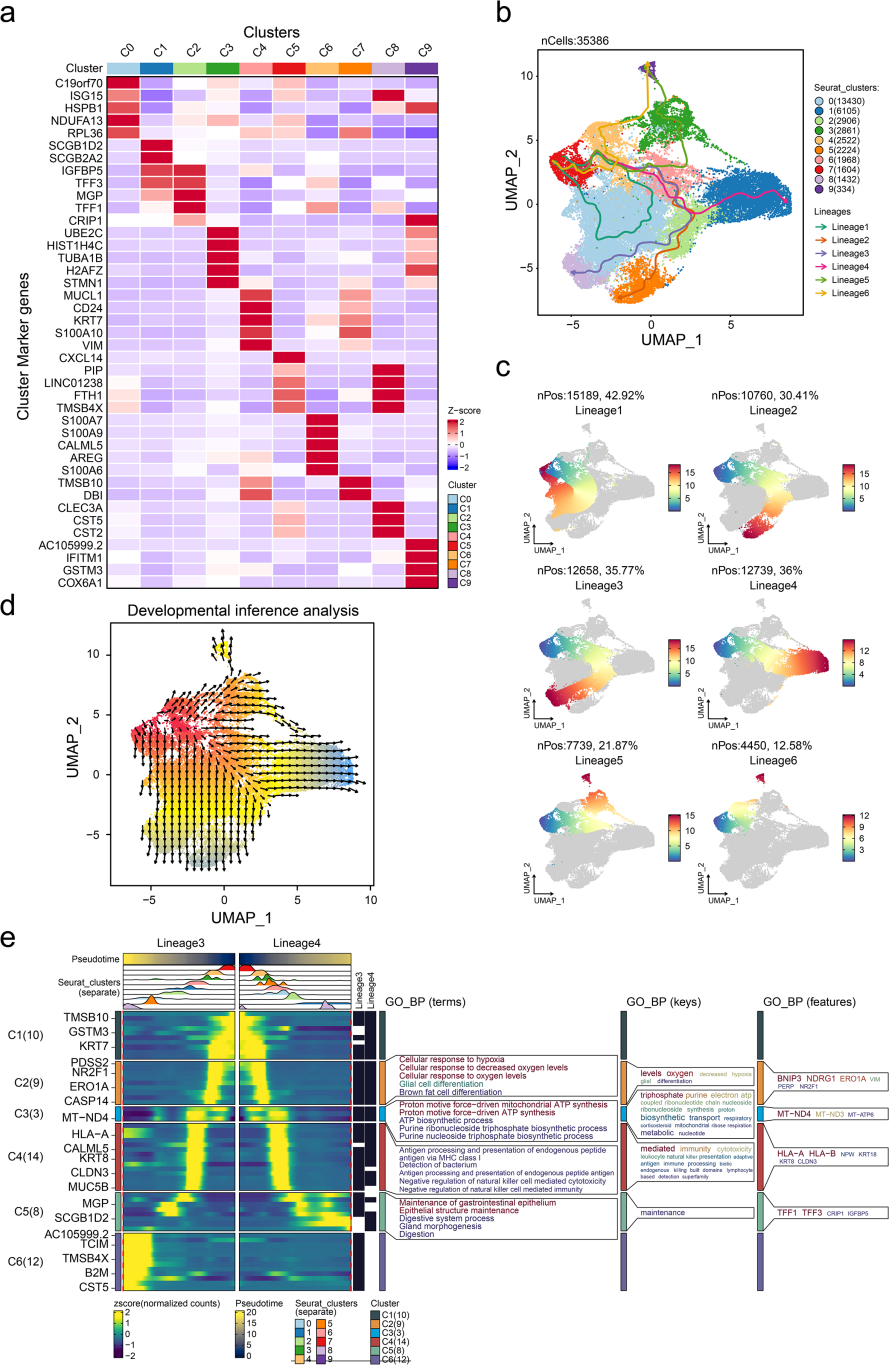
Characterization of cell trajectory of tumor cells. **(a)** Heatmap showing the overexpressed gene expression level of each tumor subgroup. Pseudotime cell trajectory of tumor cells **(b)** and six potential cell lineages **(c)**. **(d)** Cell developmental analysis confirmed the origin of cell states. **(e)** Dynamic gene expression heatmap showing the gene expression tendency based on lineages 3 and 4. GO_BP, gene ontology biological process.

Deciphering the intrinsic lineage dynamics of cell clusters is crucial to elucidate their multifaceted roles in tumor microenvironment (TME) remodeling. Thus, we analyzed the landmark genes with the highest expression in 10 different cell populations and presented them in the form of heat maps (**Figure 3A**). Cell trajectory analysis indicated six potential cell lineages that existed in these 10 clusters (**Figure 3B**), and each trajectory was displayed separately with the pseudo-time development (**Figure 3C**). Then, cell development analysis was conducted for validation, showing that lineage 4 and lineage 7 were the differentiation starting point of cell development, which is consistent with the results of the pseudo-temporal analysis. Meanwhile, we presented the trends of cell differentiation and development (**Figure 3D**). We note that C4 is the branching point of all phylogenetic evolutionary trajectories, suggesting that this subgroup has a high degree of phylogenetic plasticity. Dynamic cell trajectory analysis was also performed to confirm the changes of biological function and markers (**Figure 3E**). Collectively, our findings delineate the cellular ontogeny of ER+ cells and their associated transcriptional reprogramming during tumor progression. The identified molecular signatures may serve as diagnostic biomarkers for ER+ patients’ subpopulations and novel therapeutic targets.

### MUCL1(+) CD24(+) subcluster was correlated with prognostic outcomes of ER+ breast cancer patients

**Figure 4:**
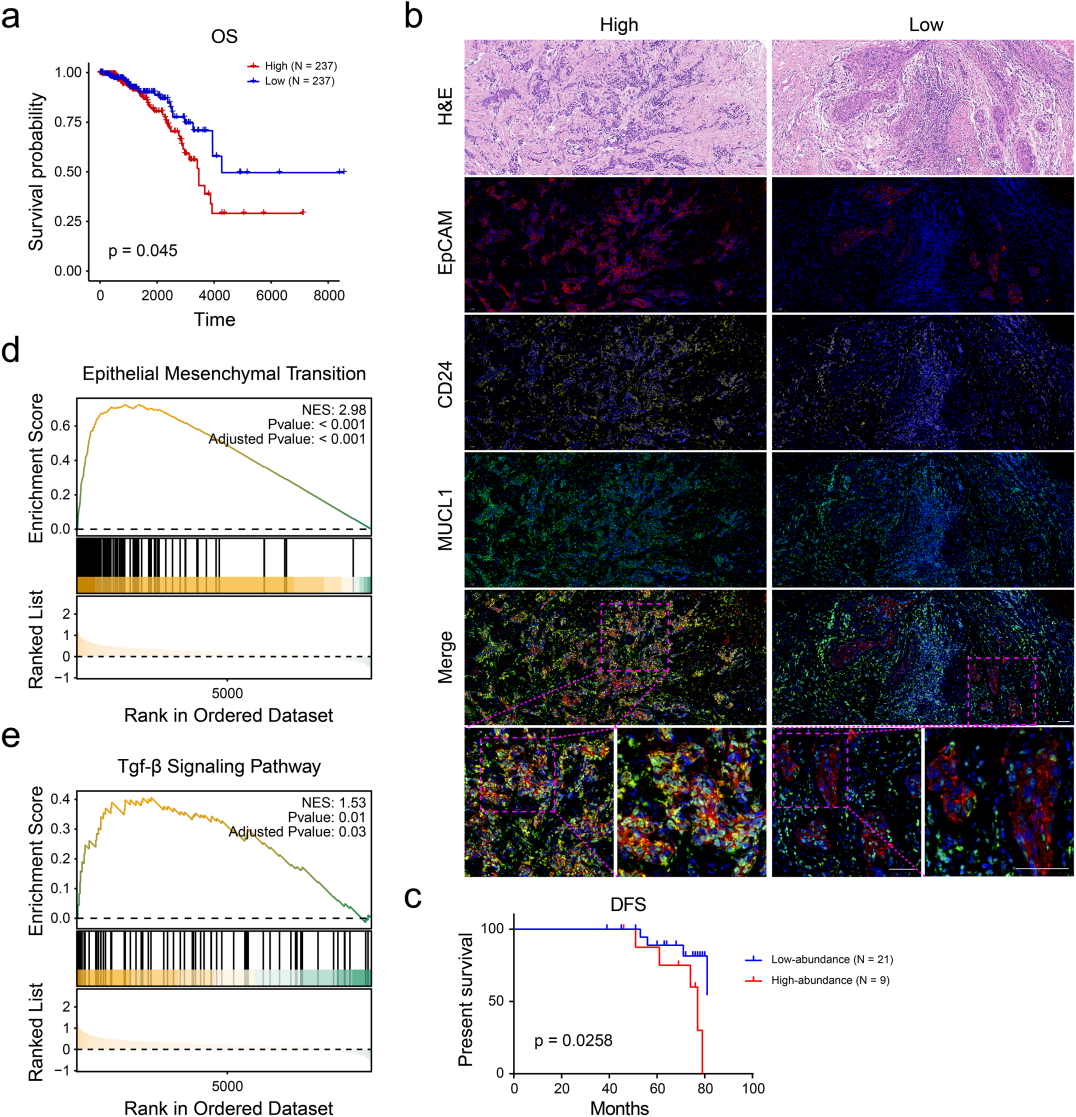
CD24(+) MUCL1(+) cells were associated with unfavorable survival of ER+ breast cancer patients. **(a)** Kaplan–Meier curve showing the survival probability of high and low abundance of C4 subgroup according to the median value of enrichment score. **(b)** Multiplex immunohistochemistry indicating the number of CD24(+) MUCL1(+) cells in ER+ breast cancer tissues. **(c)** Disease-free survival outcome of high- and low- abundance of CD24(+) MUCL1(+) cells. **(d, e)** Gene set enrichment analyses suggesting that the high abundance of CD24(+) MUCL1(+) cells was associated with epithelial–mesenchymal transition and TGF-beta signaling pathways.

Survival analysis using the KM-plotter platform revealed significant prognostic differences among breast cancer subclusters. Patients in the C4 subgroup exhibiting high invasiveness demonstrated markedly reduced overall survival (OS), suggesting aggressive clinical behavior (**Figure 4A**). Based on the analysis results in the previous study (**Figure 3A**), the markers of the C4 subgroup were identified as MUCL1, CD24, KRT7, S100A10, and VIM (**Supplementary Table S1**). A multiplex immunohistochemistry (mIHC) was performed on tumor specimens from ER+ breast cancer patients and based on the expression levels of C4-specific markers (MUCL1 and CD24); these cases were stratified into C4-high invasive and C4-low invasive subgroups (**Figure 4B**). Subsequently, we evaluated the differences in DFS between the two groups of people, the results of which indicated that the population with a high expression of MUCL1 and CD24 had a worse prognosis (**Figure 4C**). Besides that, the GSEA enrichment analysis results of the two groups of people showed that high expressions of MUCL1 and CD24 were positively associated with the EMT process (**Figure 4D**) as well as the TGF-β signaling pathway (**Figure 4E**). These findings demonstrate that the C4 subgroup, characterized by dual MUCL1(+) CD24(+) expression, correlates with aggressive tumor behavior and inferior clinical outcomes.

### The somatic mutations were associated with the upregulation of MUCL1(+) CD24(+) cells

**Figure 5:**
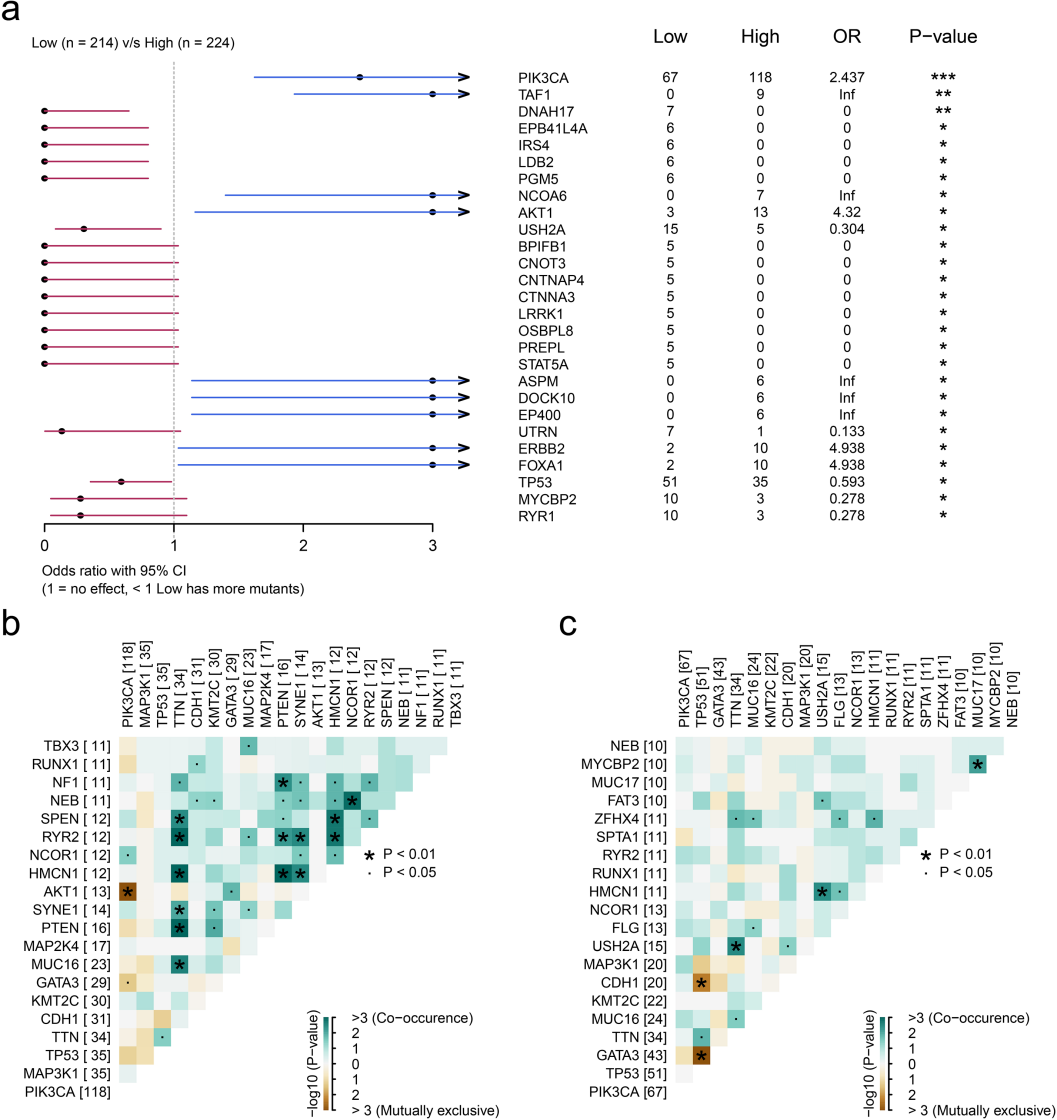
Genomic landscape of high and low infiltration levels of CD24(+) MUCL1(+) cells. **(a)** Forest plot illustrating the focal somatic single-nucleotide variant (SNV) being significantly different between patients with high and low CD24(+) MUCL1(+) cell infiltrations. OR >1 indicates a higher SNV frequency in the high CD24(+) MUCL1(+) cell infiltration group. OR <1 indicates a higher SNV frequency in the low infiltration group. *P < 0.05, **P < 0.01, ***P < 0.001. **(b, c)** Landscape of gene co-mutations in patients with low and high abundance of CD24(+) MUCL1(+) cells.

Given the established role of somatic mutations in modulating cellular infiltration patterns, we propose that tumor-derived genetic alterations may similarly regulate MUCL1(+) CD24(+) cell expression within the tumor microenvironment. To identify the most important somatic mutations related to the infiltration of MUCL1(+) CD24(+) cells, we grouped the patients into high- and low-MUCL1(+) CD24(+) groups as previously described. The SNV analysis showed that the somatic mutations of PIK3CA (*P* < 0.001), TAF1 (*P* < 0.01), and AKT1 (*P* < 0.05) were closely associated with the high-MUCL1(+) CD24(+) group (**Figure 5A**). Notably, the MUCL1(+) CD24(+) high-expression group exhibited significant genetic interaction patterns, with strong co-occurrence and mutual exclusivity relationships among key alterations (**Figure 5B**). In contrast, these patterns were markedly attenuated in the low-expression cohort (**Figure 5C**). These results reveal a subtle relationship between MUCL1(+) CD24(+) cells and somatic mutations.

### MUCL1(+) CD24(+) subcluster demonstrated association with immune response modulation

**Figure 6:**
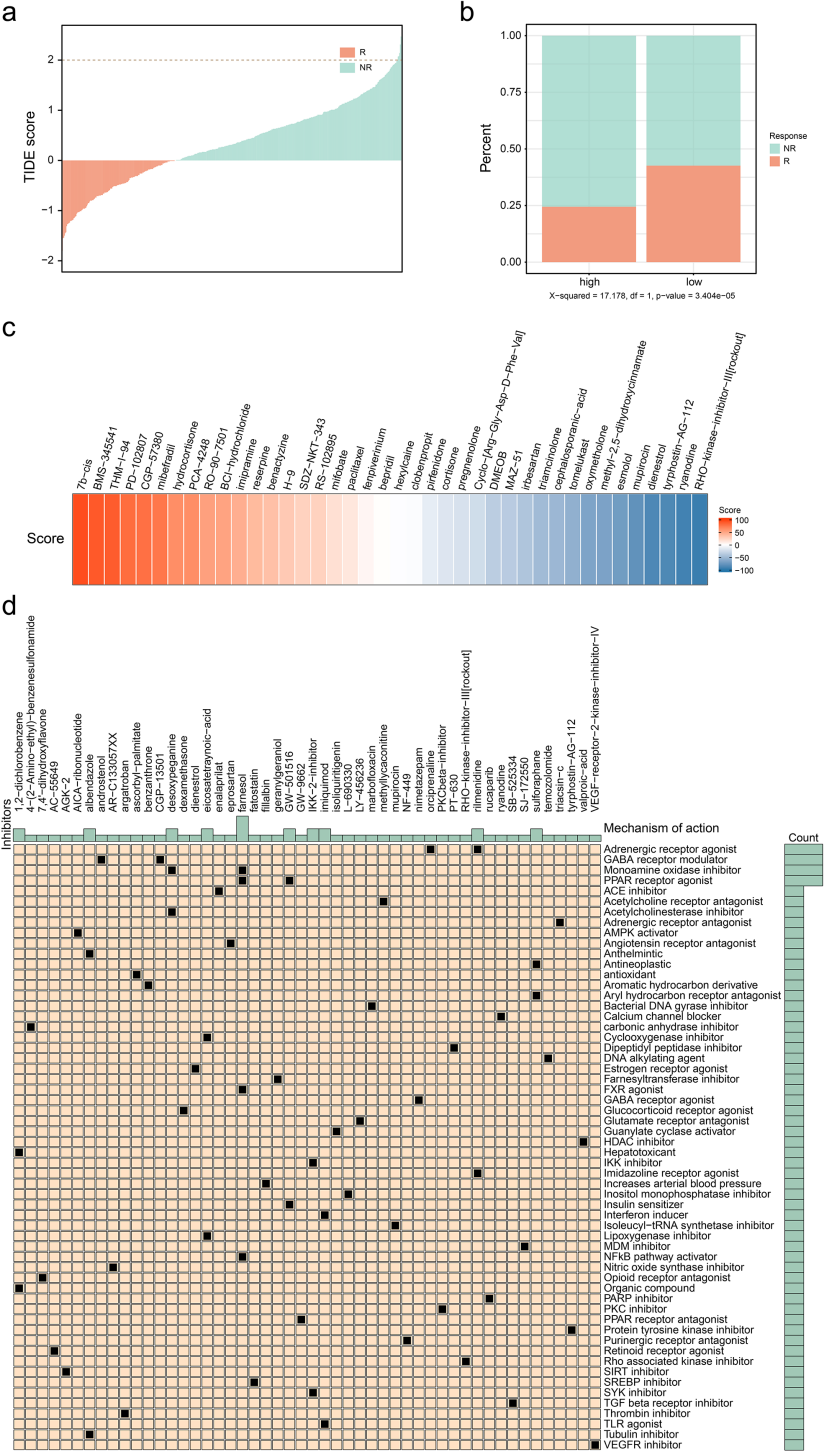
Exploration of potential therapies for patients with high infiltration level of CD24(+) MUCL1(+). **(a)** Correlation between TIDE score and immune response status. **(b)** Correlation between different C4 groups and immune response status. **(c)** Heatmap showing several results of CMAP with specific scores of drugs. **(d)** Potential drugs identified for patients with a high infiltration level of C4.

The previous study expounded that C4 subgroup cells may have a prognostic predictive role in ER+ breast cancer. Subsequently, we explored the performance of this type of subgroup in immune response and breast cancer treatment. The immune escape ability of the ER+ breast cancer cell population was evaluated by using the TIDE database (http://tide.dfci.harvard.edu/) (**Figure 6A**), and there were significant differences in the immune response ability in the groups with high and low infiltration of the C4 subpopulation, the results of which indicated that a higher percentage of C4 subpopulation exhibited a lower immune response (**Figure 6B**). Then, drugs with high sensitivity to the C4 subgroup were screened out based on the scores. The top-ranked ones include 7b-cis, BMS-345541, and THM-I-94 (**Figure 6C**). Meanwhile, the included drugs and their potential mechanisms are listed in **Figure 6D**. These findings revealed that the C4 subgroup is also involved in immune response and drug sensitivity regulatory process. Further research is conducive to providing new targets and treatment strategies for clinical practice.

### Noninvasive MRI radiomics could be a promising tool to evaluate the infiltration of MUCL1(+) CD24(+) subcluster in ER+ breast cancer

**Figure 7:**
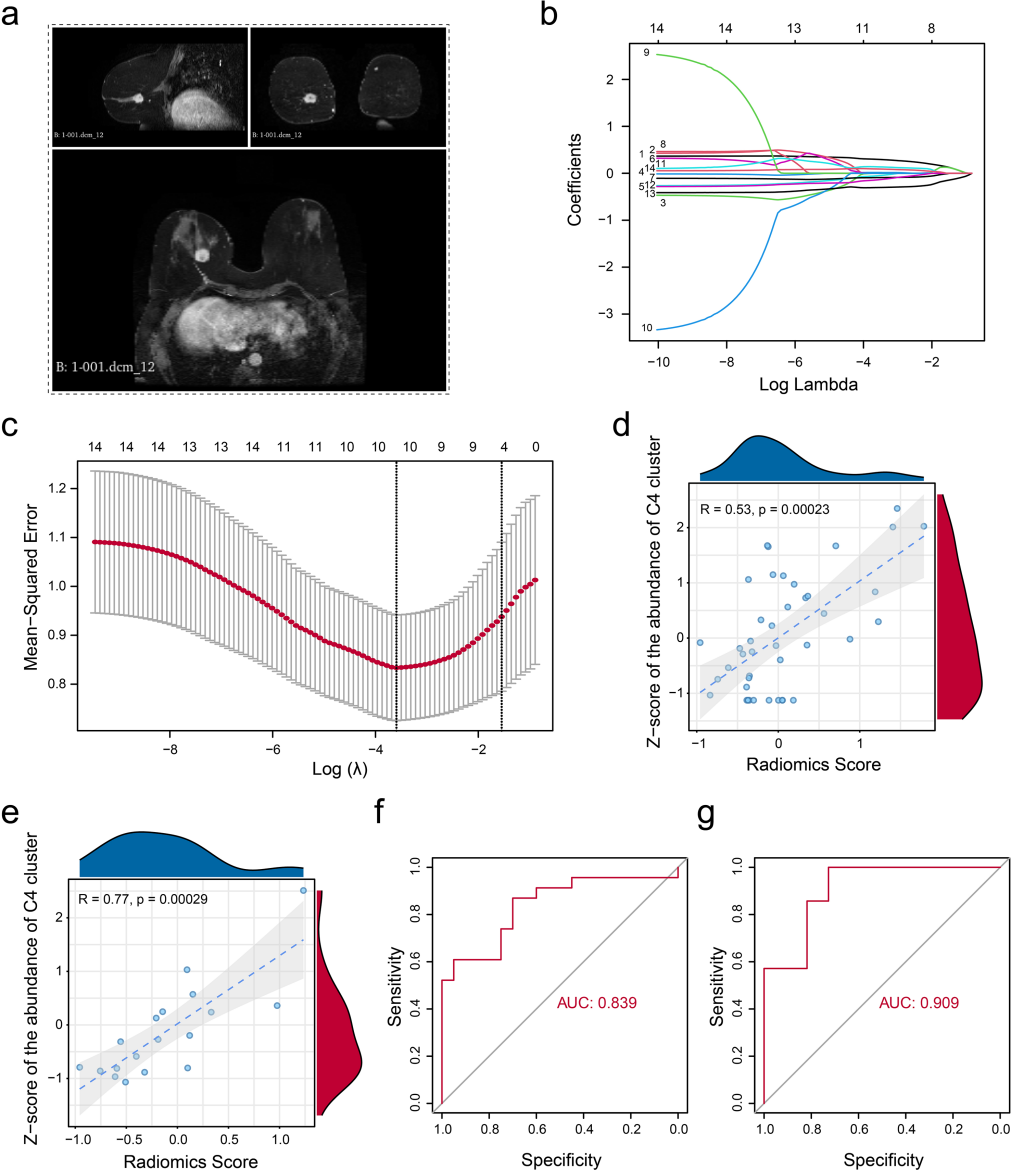
Non-invasive radiomic model construction. **(a)** Example indicating the segmentation of gross tumor volume on DCE-T1 MRI. **(b)** Parameter tuning plot for the LASSO regression analysis. **(c)** Distribution of coefficients for variables in the LASSO regression is presented, with each curve representing a radiomics feature filtered using Pearson’s correlation. **(d, e)** Pearson’s correlation was calculated in the training set **(d)** and the validation set **(e)** between the z-score-normalized abundance of CD24(+) MUCL1(+) cells assessed by the transcriptome and the fitted value obtained from the linear regression radiomics model. **(f, g)** ROC curve indicating the model’s ability to discriminate the abundance of CD24(+) MUCL1(+) cells in both the training set **(f)** and the validation set **(g)**.

Given the significant clinical associations of the MUCL1(+) CD24(+) population, we systematically evaluated its therapeutic potential applications. Our analysis included 61 ER+ breast cancer patients from the TCGA cohort with paired bulk RNA-seq and dynamic contrast-enhanced MRI (DCE-MRI) data. The tumor regions were segmented using the radiomics module in 3D Slicer software (**Figure 7A**). Pearson correlation analysis was performed to further identify significant radiomic features associated with the abundance of MUCL1(+) CD24(+) population (**Supplementary Table S2**). Then, we employed the LASSO regression algorithm based on 14 valuable features to develop a predictive model to estimate MUCL1(+) CD24(+) subpopulation abundances, balancing the feature selection with regularization to optimize model performance (**Figures 7B, C**, **Supplementary Table S3**). The cohort was randomly divided into the training set (*N* = 43) and the validation set (*N* = 18). Then, the analysis revealed that the infiltration level of the C4 cluster and the radiomics score showed a strong positive correlation in both the training set (**Figure 7D**) and the validation set (**Figure 7E**). Besides that, the exploratory analysis based on ROC curve also showed a potential association between radiomic score and the infiltration of C4 cluster infiltration in both the training set (**Figure 7F**) and the validation set (**Figure 7G**). In summary, we established a clinically applicable radiomics model that accurately predicts C4 cluster abundance, offering a non-invasive approach to personalize therapy for ER+ breast cancer patients.

## Discussion

This investigation delineates a distinct C4 cellular subpopulation within ER+ breast cancer, identified via single-cell transcriptomic profiling and defined by the co-expression of MUCL1 and CD24. This phenotype demonstrated a significant correlation with adverse clinical outcomes, prompting further interrogation of its biological determinants. We also explored the dynamic evolution of different clusters to reveal the characteristics of each subtype. Additionally, we analyzed potential somatic mutations linked to the infiltration of these clusters. Finally, a radiomic model was established to estimate the abundance of target C4 cluster. To our knowledge, this is the first study to characterize the role of MUCL1(+) CD24 (+) cells in ER+ breast cancer using multi-omics strategies, including spatial transcriptomics and radiomics. Our research provides pioneering insights into the pro-tumor effects and potential clinical applications of MUCL1(+) CD24 (+) cells in ER+ breast cancer.

Single-cell RNA sequencing has opened up new avenues for the development of tumor markers, and a large number of studies have focused on the subpopulation analysis of the tumor microenvironment—for instance, Ma et al. (20) identified a distinct luminal subgroup with a high expression level of HPN to diagnose and stratify early-stage prostate cancer by tissue-based single-cell RNA sequencing. Yang et al. (21) found a CEBPB+ tumor subcluster that specifically drives the formation of M2 tumor-associated macrophages to promote malignancy growth in glioblastoma. Additionally, Guo et al. (22) discovered that a metastasis-associated cell cluster overexpressed RAB13 in ovarian cancer by analyzing the primary and pair lymph metastatic node tissues. These studies demonstrate the great potential of single-cell sequencing in the development of tumor biomarkers. It is worth noting that studies analyzing subgroups of breast cancer have also been reported. Wang et al. (15) identified a tumor subgroup that overexpressed NENF, which is associated with distant metastasis of triple-negative breast cancer. Two distinct molecular subtypes of breast cancer stem cells have also been reported by analyzing single-cell RNA data (23). However, few studies have focused on the tumor heterogeneity of ER+ breast cancer. Our study reported a tumor cluster (C4 subgroup) with double-positive status of CD24 and MUCL1 in ER+ breast cancer, which was strongly associated with tumor metastasis. We noted that the C4 subgroup is at the differentiation bifurcation point between primary ER+ breast cancer and lymph node metastases. More interestingly, cell preference analysis showed that the C4 subgroup was enriched in primary tumor tissue, which suggests that the C4 subgroup may be the pre-differentiation state of lymph node metastatic tumor cells, showing a high degree of lineage plasticity. Furthermore, survival analysis and mIHC confirmed the unfavorable role of the C4 subgroup. The tumor metastasis-related signaling pathways including epithelial–mesenchymal transition (24) and TGF-beta signaling (25) were proved to be highly enriched in high abundance of C4 subgroup patients, supporting the pro-metastasis role of C4 subgroup. In summary, our findings reveal and define a class of tumor subpopulations that promote the metastasis of ER+ breast cancer, providing new biomarkers for the diagnosis and treatment of ER+ breast cancer.

The heterogeneity of tumor cell infiltration has been confirmed to be associated with focal somatic mutations (13). We observed that the high infiltration level of the C4 subgroup was associated with the somatic mutations of PIK3CA and FOXA1. PIK3CA-mutated ER+ metastatic breast cancer patients have been reported to demonstrate a poor outcome and resistance to chemotherapy (26). Meanwhile, FOXA1 mutations were confirmed to be associated with a lower response to aromatase inhibitors (27). These results reveal the source of infiltration heterogeneity in the C4 subpopulation and potential targeted therapeutic strategies for the C4 subpopulation. We next explore the novel therapy treatment for the C4 subgroup. We found that patients with high abundance of the C4 subgroup presented a lower proportion of immune responses by *in silico* analysis, indicating that immunotherapy may not be suitable for patients with a high infiltration of the C4 cluster. We utilized the CMAP database for the identification of potential inhibitors to target the C4 subgroup, which provides a theoretical basis for individual treatment for ER+ breast cancer.

Non-invasive assessment of radiomics has also been applied to a variety of tumors (28, 29)—for example, Wang et al. (13) used single-cell RNA to confirm the favorable role of gamma-delta T cells and developed a radiomic score to evaluate the infiltration level of gamma-delta T cells and the application of radiomics. In this study, we constructed a radiomic model to estimate the abundance of the MUCL1(+) CD24(+) subcluster. Both the training set (AUC = 0.839) and the validation set (AUC = 0.909) demonstrated a good discriminatory ability in identifying the abundance of the C4 subgroup. Overall, we constructed a model for noninvasive assessment of C4 subset abundance based on the imaging features of the C4 subset, which has good efficacy and may serve as a potential tool for future clinical translational applications.

It needs to be clarified that there are also deficiencies in our research. While our multi-omics integration provides comprehensive insights, cross-platform validation using alternative sequencing technologies (e.g., single-nuclei RNA-seq, spatial proteomics) would strengthen the findings. Additionally, *in vivo* and *in vitro* experiments need to be conducted to further explore the molecular function of the MUCL1(+) CD24(+) tumor cluster. Future work should incorporate functional validation through mechanistic studies and expand clinical correlation using independent cohorts. Although the current radiomic analysis serves as a proof of concept, prospective collection of multicenter MRI datasets will be essential for clinical translation.

## Data Availability

The datasets presented in this study can be found in online repositories. The names of the repository/repositories and accession number(s) can be found in the article/**Supplementary Material**.
